# Supplementary material for: Transformation of amorphous calcium phosphate to bone-like apatite
Source: Nat Commun. 2018 Oct 9;9:4170. doi: 10.1038/s41467-018-06570-x (PMC6177403; doi:10.1038/s41467-018-06570-x)
Supplement: Supplementary file 1 — Supplementary Information [file 41467_2018_6570_MOESM1_ESM.pdf]

Supplementary Information for

Transformation of Amorphous Calcium Phosphate to Bone-like Apatite

Antiope Lotsari[1,2], Anand K. Rajasekharan[1], Mats Halvarsson[2], Martin Andersson[1]

[1] Department of Chemistry and Chemical Engineering, Chalmers University of Technology, SE-412 96 Gothenburg, Sweden
[2] Department of Physics, Chalmers University of Technology, SE-412 96 Gothenburg, Sweden

Supplementary Figures

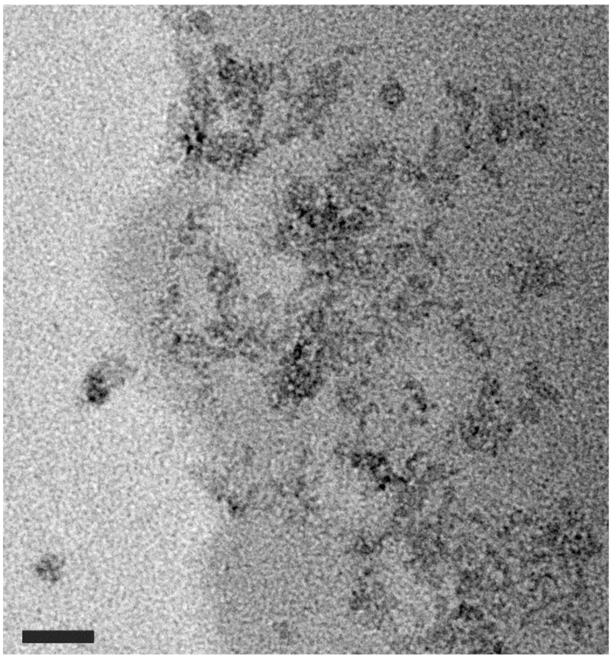

Supplementary Figure 1. Network formation of nm sized particles. The sample was aged for 1 week. Scale bar, 50 nm.

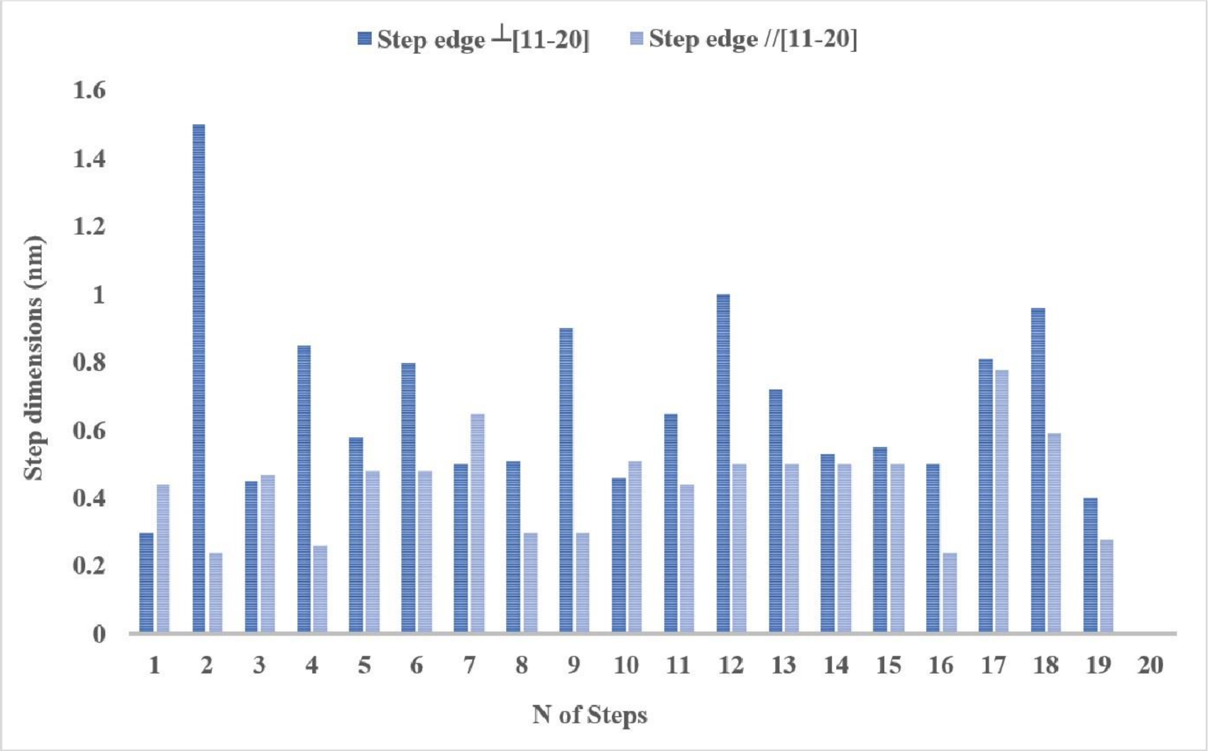

Supplementary Figure 2. Step dimensions along and perpendicular to $[11\bar{2}0]$ direction.

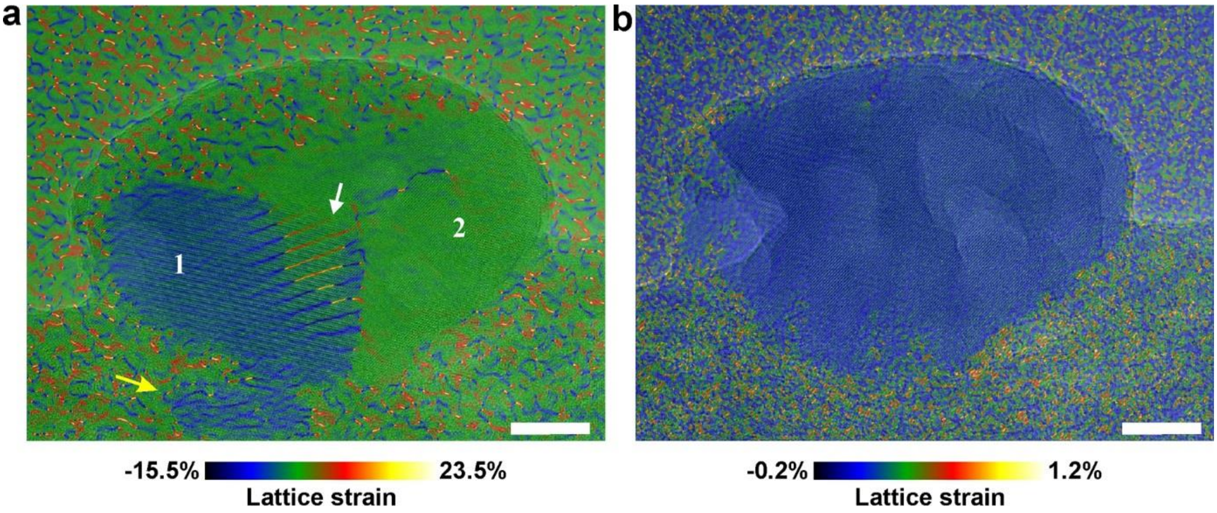

Supplementary Figure 3. Images showing the change in crystal orientation. **a** Lattice strain map obtained by GPA using the **g =** $10\bar{1}2$ spatial frequencies from area 2 superimposed on the HRTEM image of the particle depicted in Fig. 2c in which the two crystal domains with different orientations can be distinguished. The white arrow denotes an artefact due to the presence of Moiré fringes. The strain map also revealed the presence of an adjacent new crystallite along the same orientation as in area 1 (yellow arrow). **b** HRTEM of the same crystallite after 2 min and GPA lattice strain image superimposed using the same spatial frequencies. The crystal orientation from area 2 in **a,** has advanced to the whole particle which is now viewed along $[10\overline{11}]$ zone axis. Scale bars, 10 nm.

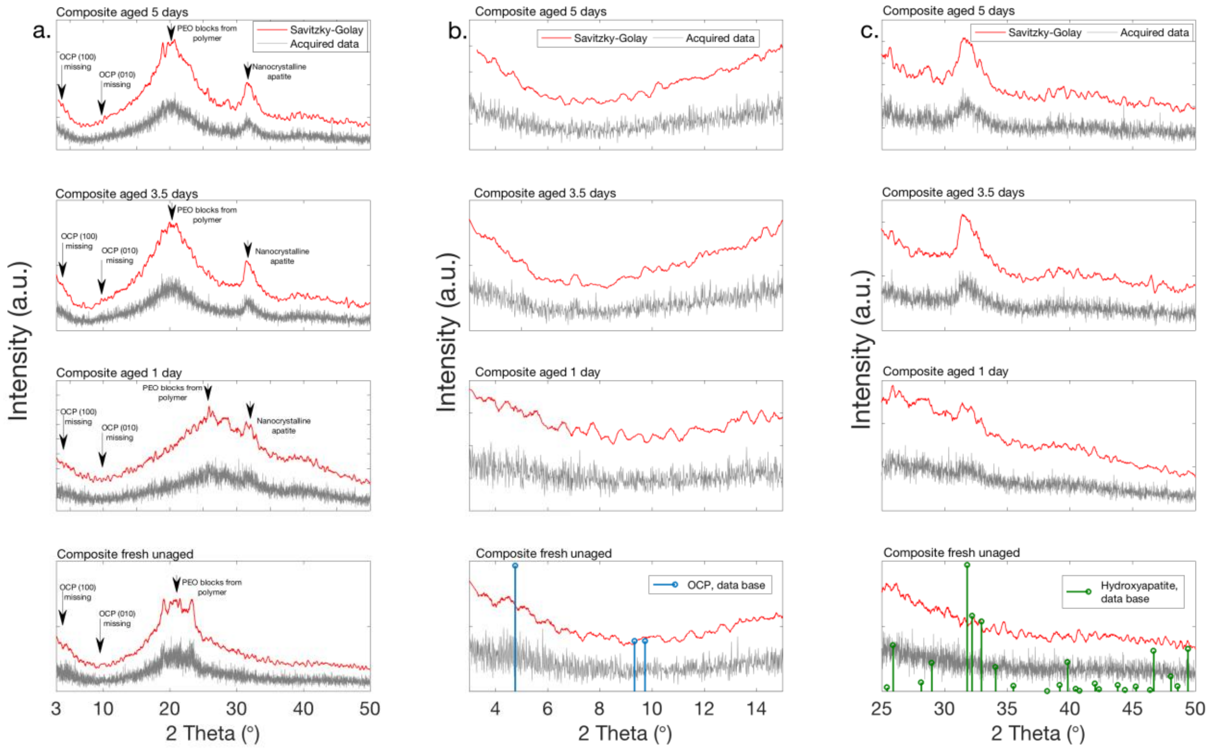

Supplementary Figure 4. XRD diffractograms of the PLLC-apatite composite. **a** XRD data shows original and Savitzky-Golay filtered data of the evolution of apatite from amorphous samples during aging of the composite. Note the absence of the (100) and (010) OCP peaks at 2 Theta 4.747° and 9.744° respectively. **b-c** XRD data of the specific regions from **a** with superimposed OCP and Hydroxyapatite data base.
